# *Drosophila* Rabex-5 restricts Notch activity in hematopoietic cells and maintains hematopoietic homeostasis

**DOI:** 10.1242/jcs.174433

**Published:** 2015-12-15

**Authors:** Theresa A. Reimels, Cathie M. Pfleger

**Affiliations:** 1 Department of Oncological Sciences, The Icahn School of Medicine at Mount Sinai, New York, NY 10029, USA; 2 The Graduate School of Biomedical Sciences, The Icahn School of Medicine at Mount Sinai, New York, NY 10029, USA

**Keywords:** Rabex-5, RabGEF1, Ras, Notch, *Drosophila* hematopoiesis, Leukemia, Hemocyte, Crystal cell, Lamellocyte, Lymph gland, Melanotic mass

## Abstract

Hematopoietic homeostasis requires the maintenance of a reservoir of undifferentiated blood cell progenitors and the ability to replace or expand differentiated blood cell lineages when necessary. Multiple signaling pathways function in these processes, but how their spatiotemporal control is established and their activity is coordinated in the context of the entire hematopoietic network are still poorly understood. We report here that loss of the gene *Rabex-5* in *Drosophila* causes several hematopoietic abnormalities, including blood cell (hemocyte) overproliferation, increased size of the hematopoietic organ (the lymph gland), lamellocyte differentiation and melanotic mass formation. Hemocyte-specific *Rabex-5* knockdown was sufficient to increase hemocyte populations, increase lymph gland size and induce melanotic masses. *Rabex-5* negatively regulates Ras, and we show that Ras activity is responsible for specific *Rabex-5* hematopoietic phenotypes. Surprisingly, Ras-independent Notch protein accumulation and transcriptional activity in the lymph gland underlie multiple distinct hematopoietic phenotypes of *Rabex-5* loss. Thus, *Rabex-5* plays an important role in *Drosophila* hematopoiesis and might serve as an axis coordinating Ras and Notch signaling in the lymph gland.

## Abstract (precis)

**Summary:** Ras regulator Rabex-5 restricts Notch during *Drosophila* embryonic and larval hematopoiesis to maintain hematopoietic homeostasis and might serve as an axis to coordinate Ras and Notch signaling.

## INTRODUCTION

*Drosophila melanogaster* has served as a genetic model for studying signaling mechanisms controlling hematopoietic processes (Dearolf, 1998; Evans et al., 2003; Jung et al., 2005; Martinez-Agosto et al., 2007; Crozatier and Vincent, 2011) for several decades. Regulation of hematopoiesis in *Drosophila* and mammals is similar; conserved pathways and transcription factors act in spatially and temporally distinct phases to ensure correct development and function of the hematopoietic system. Whereas hematopoietic cell types differ between *Drosophila* and mammals, the regulation and activity of signaling pathways is highly conserved across species.

*Drosophila* blood cells, collectively known as hemocytes, arise from a common, multipotent progenitor population called prohemocytes in two waves of hematopoiesis: first during embryonic development and second during larval development. Prohemocytes differentiate into three distinct lineages: plasmatocytes, crystal cells and lamellocytes. Plasmatocytes are present at all stages of *Drosophila* development and constitute 95% of hemocytes; they perform many functions of mammalian macrophages, as well as secrete cytokine-like molecules and antimicrobial peptides. Crystal cells are also present at all stages (Ghosh et al., 2015) and comprise 5% of hemocytes; they function in wound healing and the insect-specific immune process of melanization. Lamellocytes, a large and adherent cell type, only differentiate in the larval stage in response to large pathogens, wounding and tissue overgrowth. They do not appear in unchallenged, wild-type larvae (Rizki and Rizki, 1992; Lanot et al., 2001; Sorrentino et al., 2002; Markus et al., 2005; Pastor-Pareja et al., 2008).

In the larval stages, hemocytes exist in three compartments: the hematopoietic organ known as the lymph gland, sessile islets under the cuticle and the circulating hemolymph. The lymph gland is a series of bilateral lobes flanking the dorsal vessel. Hemocytes mature in the anterior-most pair of lobes, referred to as the primary lobes, whereas the subsequent secondary lobes of the lymph gland are primarily reservoirs of undifferentiated prohemocytes. Under normal conditions, hemocytes from the lymph gland are not released into the hemolymph until metamorphosis (Lanot et al., 2001; Holz et al., 2003; Grigorian et al., 2011a).

Ras signaling plays important roles in *Drosophila* hematopoiesis. *Heartless* (*htl*, an FGFR homolog) signaling is required for lymph gland progenitor development (Mandal et al., 2004; Grigorian et al., 2011b; Dragojlovic-Munther and Martinez-Agosto, 2013). Increased Ras activity causes hemocyte overproliferation and melanotic masses but is insufficient for crystal cell specification (Asha et al., 2003; Zettervall et al., 2004).

Rabex-5 (also called RABGEF1) negatively regulates Ras by promoting Ras ubiquitylation causing its relocalization to an endosomal compartment (Xu et al., 2010; Yan et al., 2010). We demonstrate here that loss of *Rabex-5* affects both hematopoietic waves and results in a number of hematopoietic abnormalities including increased hemocyte numbers, increased size of the larval lymph gland, lamellocyte differentiation and formation of melanotic masses. Surprisingly, Ras dysregulation did not promote all of these abnormalities. We discovered an increase in the accumulation of Notch protein and Notch transcriptional activity upon loss of *Rabex-5* in the lymph gland. Genetic interactions indicate that increased Notch activity is functionally relevant to *Rabex-5* crystal cell, larval lethality, melanotic mass, lamellocyte differentiation and lymph gland size phenotypes. Thus, we identify *Rabex-5* as a negative regulator of Notch activity in the lymph gland with a role in blood cell progenitors in order to restrict Notch activity to ensure appropriate proliferation and differentiation of specific hemocyte lineages. Given that the interaction between Ras and Notch is synergistic or antagonistic depending on the developmental context, a role for *Rabex-5* in the regulation of both Notch and Ras might elucidate how these complicated relationships are coordinated.

## RESULTS

### *Rabex-5* is required in *Drosophila* blood cells to prevent melanotic masses

**Fig. 1.**
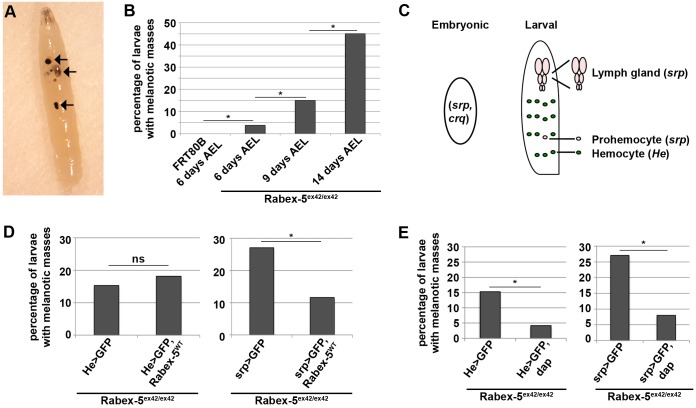
***Rabex-5* is required in blood cells to prevent melanotic mass formation.** (A) Melanotic masses in larvae homozygous for the deletion allele *Rabex-5^ex42^* (arrows; anterior, top). (B) At least one melanotic mass was seen in 3.8% of *Rabex-5^ex42/ex42^* larvae compared to 0% in control larvae 6 days AEL. Incidence of melanotic masses in *Rabex-5^ex42/ex42^* larvae increased to 15% 9 days AEL and 45% 14 days AEL. (C) *Serpent*- (*srp*) and *croquemort*- (*crq*) *gal4* express in embryonic hemocytes. *Srp-gal4* also expresses in all hemocytes of the larval lymph gland and in circulating prohemocytes. *Hemese*- (*He*) *gal4* expresses in larval hemocytes. (D) Expressing wild-type *Rabex-5* using *srp-gal4*, but not *He-gal4*, in a *Rabex-5^ex42/ex42^* background (*Rabex-5^ex42/ex42^*; *srp*>GFP, *Rabex-5^WT^* and *Rabex-5^ex42/ex42^*; *He>GFP, Rabex-5^WT^*) decreased the incidence of melanotic masses compared to those in controls (*Rabex-5^ex42/ex42^*; *srp>GFP* and *Rabex-5^ex42/ex42^*; *He>GFP*) 14 days AEL. (E) Expressing *dap* using either *He-gal4* or *srp-gal4* in a *Rabex-5^ex42/ex42^* background (*Rabex-5^ex42/ex42^*; *He>GFP, dap* and *Rabex-5^ex42/ex42^*; *srp>GFP, dap*) decreased the incidence of melanotic masses compared to those in controls (*Rabex-5^ex42/ex42^*; *He>GFP* and *Rabex-5^ex42/ex42^*; *srp>GFP*) 14 days AEL. ^*P*≤0.05, *P≤0.01.

We previously reported melanotic mass formation (Fig. 1A), and larval and pupal lethality in *Drosophila* that lack the neoplastic tumor suppressor *Rabex-5* (Yan et al., 2010). At least one melanotic mass was found in 3.8% of larvae homozygous for the deletion allele *Rabex-5^ex42^* (referred to as *Rabex-5*-null larvae) 6 days after egg laying (AEL). The incidence of melanotic masses increased over time to 45% 14 days AEL (Fig. 1B). Melanotic masses were of variable size, number and location within the body cavity. In the absence of parasitization, melanotic masses are often associated with abnormalities in the hematopoietic system, including autoimmune-like responses to self-tissue and dysregulation of proliferation leading to excess hemocyte numbers (Watson et al., 1994; Asha et al., 2003; Zettervall et al., 2004; Minakhina and Steward, 2006). To establish whether there is a requirement for *Rabex-5* to prevent melanotic mass formation, we expressed wild-type *Rabex-5* (*Rabex-5^WT^*) using *Hemese-gal4* (*He-gal4*) or *Serpent-gal4* (*srp-gal4*) (Fig. 1C, Table S1). *Hemese* is a transmembrane protein expressed in all hemocyte lineages beginning in the second larval instar (Kurucz et al., 2003; Jung et al., 2005). *He-gal4* expresses in ∼70% of circulating hemocytes, in sessile hemocytes and at low levels in the larval lymph gland (Zettervall et al., 2004), but does not express in the embryo. *Serpent* is a GATA family member and the earliest known transcription factor required for embryonic and larval hemocyte development (Rehorn et al., 1996; Lebestky et al., 2000). *Srp-gal4* expresses in embryonic hemocytes (Narbonne-Reveau et al., 2011) as well as in prohemocytes and all lymph gland cells of the larval stages (Jung et al., 2005). In *Rabex-5*-null larvae, expressing *Rabex-5^WT^* by using *He-gal4* (*Rabex-5^ex42/ex42^; He>Rabex-5^WT^*) did not reduce the incidence of melanotic masses observed at 14 days AEL; however, expressing *Rabex-5^WT^* by using *srp-gal4* (*Rabex-5^ex42/ex42^; srp>Rabex-5^WT^*) reduced melanotic mass formation more than twofold (Fig. 1D). This indicates a specific requirement for *Rabex-5* during hematopoiesis to prevent melanotic masses. To determine whether hemocyte overproliferation contributes to the melanotic mass phenotype, we utilized *Drosophila* cyclin-dependent kinase inhibitor *dacapo* (*dap*). In *Rabex-5*-null larvae 14 days AEL, expressing *dap* in the hematopoietic system reduced melanotic mass formation (*Rabex-5^ex42/ex42^; He>dap* and *Rabex-5^ex42/ex42^; srp>dap*, Fig. 1E). Taken together, these findings suggest a role for *Rabex-5* to restrict hemocyte proliferation and prevent melanotic mass formation. *Rabex-*5-null lethality is likely to be pleiotropic; however, *He-gal4* directed *Rabex-5* expression and *He-gal4* or *srp-gal4* directed *dap* expression decreased larval lethality (Fig. S1A,B). This suggests hemocyte overproliferation also contributes to *Rabex-5*-null larval lethality.

### Homozygous loss of *Rabex-5* in *Drosophila* larvae causes hematopoietic abnormalities

**Fig. 2.**
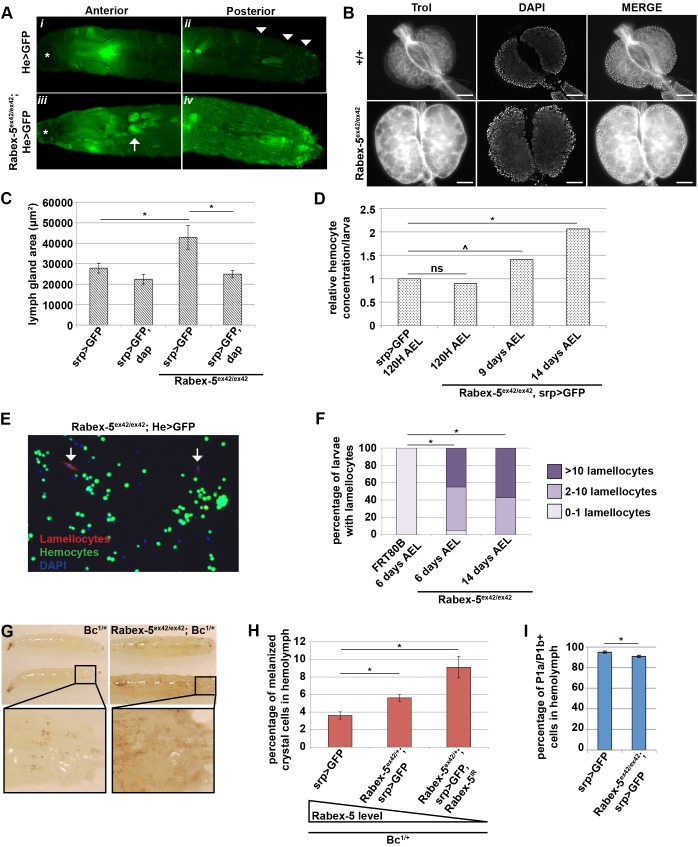
**Loss of *Rabex-5* causes a range of hematopoietic abnormalities.** (A) Wild-type (*He>GFP, i*, *ii*) and *Rabex-5^ex42/ex42^* (*Rabex-5^ex42/ex42^; He>GFP, iii*, *iv*) larvae expressing GFP in hemocytes. Arrowheads (*ii*) indicate sessile hemocytes, arrow (*iii*) indicates lymph gland, and asterisks (*i*, *iii*) mark mouth hooks for a reference point. The strong anterior signal is GFP fluorescence in the salivary glands as seen with many *Gal4* drivers. (B) Lymph glands dissected from control and *Rabex-5^ex42/ex42^* larvae 5 days AEL. The basement membrane was marked by Trol expression. Scale bars: 50 μm. (C) Compared to that in controls (*srp>GFP*), the area of the primary lymph gland lobes increased in *Rabex-5^ex42/ex42^* larvae (*Rabex-5^ex42/ex42^; srp>GFP*) but was restored with hemocyte-specific expression of *dap* (*Rabex-5^ex42/ex42^; srp>GFP, dap*). (D) At 120 h AEL, circulating hemocyte concentrations of *Rabex-5^ex42/ex42^* larvae (*Rabex-5^ex42/ex42^; srp>GFP*) were similar to controls (*srp>GFP*). Hemocyte concentrations of *Rabex-5^ex42/ex42^* larvae increased 9 days and 14 days AEL. (E) Lamellocytes were detected by using L1a, L1b and L1c antibodies in the hemolymph of *Rabex-5^ex42/ex42^* larvae (arrows, *Rabex-5^ex42/ex42^; He>GFP*). (F) Lamellocytes were detected in 95% of *Rabex-5^ex42/ex42^* larvae and 0% of controls larvae 6 days AEL. (G) Melanized crystal cells were visible in a heterozygous *Bc^1^* background (left, *Bc^1/+^*). *Rabex-5^ex42/ex42^* larvae (right, *Bc^1/+^; Rabex-5^ex42/ex42^*) showed a marked increase in the number of melanized crystal cells. (H) The percentage of melanized crystal cells in the hemolymph of larvae 120 h AEL increased with decreasing *Rabex-5* levels. (I) The percentage of circulating plasmatocytes detected by P1a and P1b antibodies decreased in *Rabex-5^ex42/ex42^* larvae (*Rabex-5^ex42/ex42^; srp>GFP*) 9 days AEL compared to that in controls (*srp>GFP*) at 120 h AEL. ^*P*≤0.05, *P≤0.01.

Because the *Rabex-5*-null melanotic mass phenotype depends on proliferation of hemocytes, we further investigated the role of *Rabex-5* within the hematopoietic system. Visualizing hemocytes *in vivo* using *He>GFP*, we observed a dramatic disruption of the hematopoietic system in *Rabex-5*-null larvae (Fig. 2A*iii*, *iv*) compared to that in controls (Fig. 2A*i*, *ii*). The hematopoietic organ, the lymph gland, became clearly visible through the cuticle of *Rabex-5*-null larvae (arrow in Fig. 2A*iii*) but not control larvae (Fig. 2A*i*). The size of *Rabex-5*-null lymph glands increased drastically (Fig. 2B,C and Fig. S2A); lymph glands became so overgrown that they dissociated from the dorsal vessel upon dissection, were morphologically unrecognizable, and/or physically indistinguishable from other overgrown tissues. This is consistent with overgrowth seen previously for *Rabex-5*-mutant epithelial tissues (Yan et al., 2010; Thomas and Strutt, 2014). *Srp-gal4* directed *dap* expression did not affect the lymph gland area in control larvae (*srp>dap*) but restored the lymph glands of *Rabex-5*-null larvae to wild-type size (*Rabex-5^ex42/ex42^; srp>dap*, Fig. 2C).

*Rabex-5*-null larvae also showed a dramatic increase in hemocyte numbers throughout the body cavity (Fig. 2A*iii*, *iv* compared to 2A*i*, *ii*). At 120 h AEL, hemocyte concentrations in *Rabex-5*-null larvae are similar to the control; hemocyte concentrations increased in *Rabex-5*-null larvae by 9 days AEL (Fig. 2D, Fig. S2B). Changes in hemocyte proportions as monitored by *srp>GFP* and *He>GFP* were also seen by 9 days AEL (Fig. S2C). These increases did not result from a ruptured or empty lymph gland because the lymph gland remained populated and the basement membrane, marked by Trol expression (Grigorian et al., 2011a), remained intact in *Rabex-5*-null larvae (Fig. 2B). Given increased hemocyte concentrations, the increase in GFP-positive hemocytes in *Rabex-5*-null larvae might result from hemocyte overproliferation or from dysregulation of hemocyte lineages and markers.

Unexpectedly, we observed lamellocytes in the hemolymph of *Rabex-5*-null larvae, detected by a mixture of L1a, L1b and L1c antibodies (Kurucz et al., 2007). Lamellocytes in wild-type larvae only differentiate in response to specific immune challenges. Despite the lack of external immune challenges sufficient to induce lamellocyte differentiation in our system, lamellocytes were observed in 95% of *Rabex-5*-null larvae, compared to 0% of control larvae 6 days AEL (Fig. 2E,F). Expression of *dap* in hemocytes did not suppress lamellocyte differentiation (Fig. S2D), suggesting that lamellocyte differentiation does not result from increased hemocyte proliferation.

To determine whether loss of *Rabex-5* affects other hemocyte lineages, we examined crystal cell and plasmatocyte populations. We utilized *Bc^1^*, an allele of *Black cells* (*Bc*) that causes spontaneous melanization of crystal cells, to visualize crystal cells *in vivo*. *Rabex-5*-null larvae (*Bc^1/+^; Rabex-5^ex42/ex42^*) showed a marked increase in the number of melanized crystal cells compared to control larvae (*Bc^1/+^*, Fig. 2G). The percentage of melanized crystal cells in the hemolymph increased with decreasing levels of *Rabex-5* (Fig. 2H). A similar ∼1.5-fold increase in the percentage of crystal cells upon loss of *Rabex-5* was confirmed by using an antibody against lozenge, a transcription factor required for crystal cell specification (Lebestky et al., 2000), and by using heat to induce melanization of crystal cells *in vivo* (Fig. S2E,F). Excess crystal cells might reflect overproliferation and release from the sessile compartment or transdifferentiation from plasmatocytes (Leitao and Sucena, 2015). The percentage of plasmatocytes present in the hemolymph, which were detected by using a mixture of P1a and P1b antibodies (Kurucz et al., 2007), decreased in *Rabex-5*-null larvae (*Rabex-5^ex42/ex42^; srp>GFP* and *Rabex-5^ex42/ex42^; He>GFP*) compared to controls (*srp>GFP* and *He>GFP*, Fig. 2I and Fig. S2G). Given that plasmatocytes have been reported to transdifferentiate to lamellocytes as well, the appearance of large numbers of lamellocytes and the increase in crystal cells might explain the decrease in circulating plasmatocytes (Honti et al., 2010; Krzemien et al., 2010; Stofanko et al., 2010). Alternatively, loss of *Rabex-5* might promote a progenitor-like state or alter gene expression patterns, such that plasmatocyte-specific epitopes are no longer present. Hemocytes overexpressing activated Ras have been reported to alter mRNA expression compared to wild-type hemocytes (Asha et al., 2003).

### *Rabex-5* is required in hemocytes to maintain hematopoietic balance

**Fig. 3.**
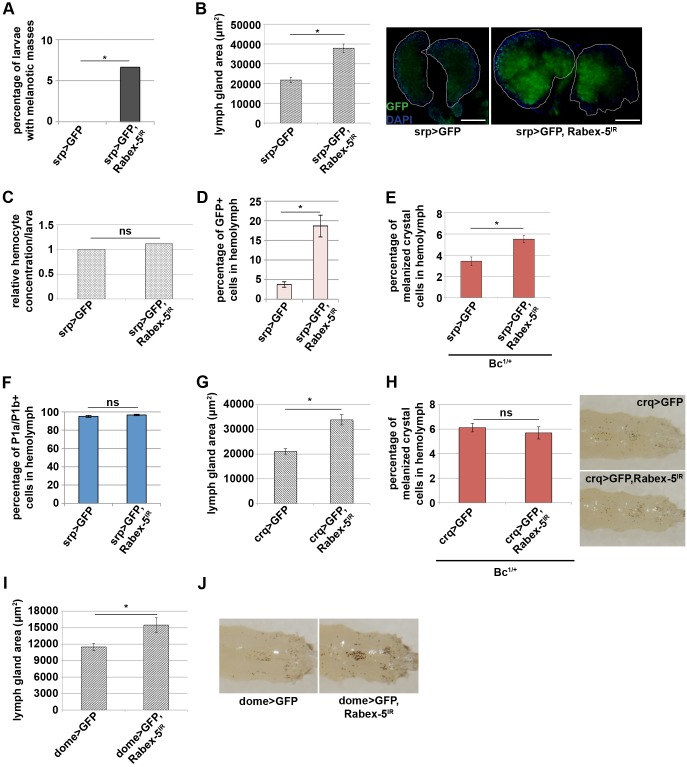
***Rabex-5* is required in blood cells to restrict proliferation, differentiation and the size of the lymph gland.** (A) *Rabex-5* RNAi (*srp>GFP, Rabex-5^IR^*) caused melanotic masses in 6.7% of larvae compared to 0% in control larvae (*srp>GFP*) 6 days AEL. (B) *Rabex-5* RNAi (*srp>GFP, Rabex-5^IR^*) increased the area of the primary lymph gland lobes compared to those in controls (*srp>GFP*) 4 days AEL. DAPI staining is shown in blue. Scale bars: 50 μm. (C) *Rabex-5* RNAi (*srp>GFP, Rabex-5^IR^*) did not change circulating hemocyte concentrations compared to those in controls (*srp>GFP*) 120 h AEL. (D) *Rabex-5* RNAi in hemocytes (*srp>GFP, Rabex-5^IR^*) increased the percentage of circulating GFP-positive hemocytes compared to that of controls (*srp>GFP*) 120 h AEL. (E) *Rabex-5* RNAi in hemocytes in a *Bc^1^* heterozygous background (*Bc^1/+^; srp>GFP, Rabex-5^IR^*) increased the percentage of melanized crystal cells in the hemolymph compared to that of controls (*Bc^1/+^; srp>GFP*) 120 h AEL. (F) *Rabex-5* knockdown by using *srp-gal4* (*srp>GFP, Rabex-5^IR^*) did not change the percentage of circulating plasmatocytes compared to that of controls (*srp>GFP*) 120 h AEL. (G) *Rabex-5* RNAi in embryonic hemocytes (*crq>GFP, Rabex-5^IR^*) increased the area of the primary lymph gland lobes compared to that in controls (*crq>GFP*) 5 days AEL. (H) *Rabex-5* RNAi in embryonic hemocytes in a *Bc^1^* heterozygous background (*Bc^1/+^; crq>GFP, Rabex-5^IR^*) did not alter the percentage of melanized crystal cells in the hemolymph compared to that in controls (*Bc^1/+^; crq>GFP*) 120 h AEL. Heat-induced melanization of crystal cells *in vivo* also showed no difference. (I) *Rabex-5* RNAi in the medullary zone of the larval lymph gland (*dome>GFP, Rabex-5^IR^*) increased the area of the primary lobes 4 days AEL and increased crystal cell numbers visualized by heating larvae (J) compared to those of controls (*dome>GFP*). *P≤0.01.

Both *srp-gal4*-directed expression of *Rabex-5* and of *dap* in *Rabex-5*-null larvae suppressed melanotic mass formation (Fig. 1D,E), indicating a requirement for *Rabex-5* specifically in the hematopoietic system and suggesting a role for *Rabex-5* to restrict hemocyte proliferation. To investigate a specific requirement for *Rabex-5* within the hematopoietic system, we performed RNA interference (RNAi) of *Rabex-5* by using *srp-gal4* and an inducible inverted repeat allele, *Rabex-5^IR^,* we characterized previously (Yan et al., 2010). Surprisingly, reducing *Rabex-5* levels by using *srp-gal4* was sufficient to cause melanotic masses in 6.7% of larvae (Fig. 3A). *Rabex-5* knockdown increased the area and the GFP intensity of the primary lymph gland lobes (Fig. 3B). Although *Rabex-5* knockdown was insufficient to increase hemocyte concentration (Fig. 3C), it was sufficient to alter circulating hemocyte proportions. Compared to that of controls, RNAi of *Rabex-5* in hemocytes increased the percentage of GFP-positive hemocytes in circulation **(**Fig. 3D) to an extent similar to that observed in *Rabex-5*-null larvae (Fig. S2C). RNAi of *Rabex-5* increased the percentage of circulating crystal cells (melanized cells, Fig. 3E) to an extent similar to that seen in *Rabex-5* heterozygous larvae (Fig. 2H). The basement membrane of lymph glands, marked by Trol expression, remained intact upon loss of *Rabex-5* (Fig. S3); the increased percentage of circulating hemocytes did not result from rupture or emptying of the lymph gland. In contrast, RNAi of *Rabex-5* did not significantly increase the percentage of plasmatocytes in circulation compared to that of controls (P1a/P1b-positive cells, Fig. 3F). These data indicate an intrinsic requirement for *Rabex-5* in the hematopoietic system in order to prevent melanotic masses, restrict proliferation in the primary lymph gland and maintain appropriate proportions of hemocytes in the hemolymph.

Hematopoiesis in *Drosophila* occurs in two waves. To determine whether *Rabex-5* is required to maintain hematopoietic balance in the embryonic wave, the larval wave or both, we used *croquemort-gal4* (*crq-gal4*) to perform RNAi of *Rabex-5* specifically in hemocytes of embryonic origin (Fig. 1C, Table S1). *Rabex-5* knockdown by using *crq-gal4* (*crq>Rabex-5^IR^*) increased the area of the primary lymph gland lobes (Fig. 3G) but did not affect crystal cell populations (Fig. 3H) or induce melanotic masses (not shown) compared to those in controls (*crq>GFP*). Similarly, we used *domeless-gal4* (*dome-gal4*) to reduce *Rabex-5* specifically in hemocytes of larval origin. *Rabex-5* knockdown by using *dome-gal4* (*dome>Rabex-5^IR^*) increased the area of the primary lobes (Fig. 3I) and increased crystal cell numbers (Fig. 3J) compared to those in controls (*dome>GFP*). These results suggest that *Rabex-5* is required during each wave of hematopoiesis but may have developmental stage-specific functions.

### Reduction of *Ras* gene dosage suppresses larval lethality and lymph gland size but not other hematopoietic abnormalities

*Rabex-5* loss in *Drosophila* was originally reported to increase both organismal and organ size, as well as to cause specification and differentiation defects, such as ectopic wing veins and eye/antennal transformations (Yan et al., 2010). These phenotypes are sensitive to *Ras* activity; reducing the gene dosage of *Ras* restored body size and wing area to those of controls, and largely suppressed the specification and differentiation defects (Yan et al., 2010). *Rabex-5* restricted ERK activation through its E3 ubiquitin ligase activity (Xu et al., 2010; Yan et al., 2010).

**Fig. 4.**
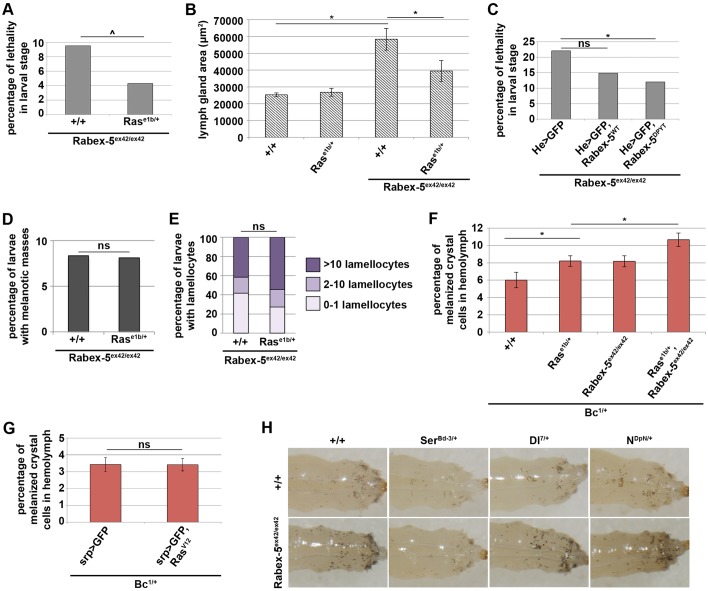
**Melanotic mass formation and lamellocyte differentiation are not sensitive to Ras gene dosage.** (A) Reducing Ras gene dosage with loss-of-function allele *Ras^e1b^* in a *Rabex-5^ex42/ex42^* background (*Rabex-5^ex42/ex42^; Ras^e1b/+^*) decreased larval lethality compared to that of controls (*Rabex-5^ex42/ex42^*). (B) Reducing Ras gene dosage restored lymph gland area in a *Rabex-5^ex42/ex42^* background compared to that in controls (*Rabex-5^ex42/ex42^; Ras^e1b/+^* and *Rabex-5^ex42/ex42^*) but had no effect on lymph gland area alone (*Ras^e1b/+^*) 5 days AEL. (C) In a *Rabex-5^ex42/ex42^* background, expressing GEF-inactive *Rabex-5* (*Rabex-5^ex42/ex42^; He>GFP, Rabex-5^DPYT^*) suppressed larval lethality, and wild-type *Rabex-5* (*Rabex-5^ex42/ex42^; He>GFP, Rabex-5^WT^*) showed a trend to suppress larval lethality compared to that in controls (*Rabex-5^ex42/ex42^; He>GFP*). Reducing Ras gene dosage in a *Rabex-5^ex42/ex42^* background (*Rabex-5^ex42/ex42^; Ras^e1b/+^*) did not affect the incidence of melanotic masses (D), the percentage of larvae containing lamellocytes (E) 14 days AEL, or the increased crystal cells (F) 9 days AEL. (G) Expressing constitutively active *Ras^V12^* in a *Bc^1^* heterozygous background (*Bc^1/+^; srp>GFP, Ras^V12^*) did not increase the percentage of melanized crystal cells in the hemolymph compared to that in controls (*Bc^1/+^; srp>GFP*) 120 h AEL. (H) Crystal cells were visualized by heating larvae 5 days AEL. Dominant-negative *Serrate* (*Ser^Bd-3/+^*) strongly suppressed the increase in the numbers of crystal cells within a *Rabex-5^ex42/ex42^* background. The loss-of-function *Delta* allele (*Dl^7/+^*) subtly suppressed the increased crystal cell numbers. Duplication of *Notch* (*N^DpN/+^*) further increased crystal cell numbers in a *Rabex-5^ex42/ex42^* background. ^*P*≤0.05, *P≤0.01.

To determine whether Ras inhibition underlies *Rabex-5*-null hematopoietic phenotypes, we reduced *Ras* gene dosage or restored the *Rabex-5* E3 ligase domain in the hematopoietic system. Reducing *Ras* gene dosage by using the loss-of-function allele *Ras^e1b^* suppressed larval lethality in *Rabex-5*-null larvae (*Rabex-5^ex42/ex42^; Ras^e1b/+^*; Fig. 4A) and restored the size of the lymph gland (Fig. 4B). To restore *Rabex-5* E3 ligase function, we used *He-gal4* in order to express either *Rabex-5^WT^* or full-length *Rabex-5* with an intact E3 ligase domain and an inactive Rab5 GEF domain (*Rabex-5^DPYT^*) that had been characterized previously (Yan et al., 2010). Expressing *Rabex-5^DPYT^* suppressed larval lethality in a *Rabex-5*-null background, and expressing *Rabex-5^WT^* showed a trend to suppress larval lethality in a *Rabex-5*-null background (Fig. 4C, Fig. S1A).

The abilities of the *Ras* mutation and the *Ras* inhibitory domain of *Rabex-5* to suppress larval lethality and to suppress increased lymph gland size are consistent with the model that increased Ras activity in the hematopoietic system mediates, in part, these phenotypes. Together with *dap*-dependent suppression of these phenotypes (Fig. 2C and Fig. S1B), this might indicate that excess proliferation due to elevated Ras activity in the hematopoietic system contributes to larval lethality and increased lymph gland size.

Reducing the *Ras* gene dosage did not suppress melanotic mass formation (Fig. 4D), lamellocyte differentiation (Fig. 4E) or the phenotype of increased crystal cell numbers (Fig. 4F) in *Rabex-5*-null larvae. Furthermore, expressing constitutively active *Ras^V12^* did not increase the percentage of circulating crystal cells (melanized cells, Fig. 4G), suggesting that melanotic mass formation, lamellocyte differentiation and increased numbers of crystal cells are not the result of increased Ras activity.

*Rabex-5* knockdown, however, was sufficient to increase the percentage of circulating crystal cells (Fig. 3E). Given the instructive role of Notch signaling in crystal cell specification (Duvic et al., 2002; Lebestky et al., 2003; Mandal et al., 2007; Krzemien et al., 2010) and the reported roles in lamellocyte differentiation (Duvic et al., 2002; Small et al., 2014), we further investigated the involvement of Notch. Encouragingly, genetic modification of certain Notch signaling components changed the *Rabex-5*-null crystal cell phenotype (Fig. 4H, summarized in Fig. 8D). The *Rabex-5*-null crystal cell phenotype was strongly suppressed by a dominant-negative allele of Notch ligand *Serrate* (*Ser*), *Ser^Bd-3^*, consistent with reported effects of this allele on crystal cells (Lebestky et al., 2003). The crystal cell phenotype was subtly suppressed by a loss-of-function allele of Notch ligand *Delta* (*Dl*), *Dl^7^*, and enhanced by *Notch* duplication (*DpN*).

### *Rabex-5* knockdown increases Notch accumulation in the larval lymph gland

**Fig. 5.**
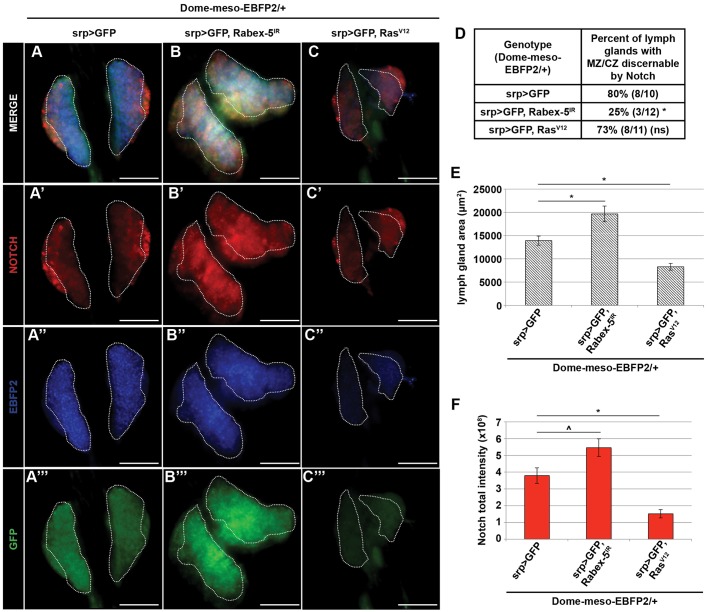
**Loss of *Rabex-5* leads to Ras-independent dysregulation of Notch protein across the primary lymph gland lobes.** (A–C) (A-C,A″-C″) Expression of EBFP2 (traced in white) marked the medullary zone (MZ) of the primary lymph gland. (A′) In control larvae (*Dome-meso-EBFP2/+; srp>GFP*) Notch expression was low in the MZ and distinct from the high expression in the outer, cortical zone (CZ). (B′) *Rabex-5* RNAi in hemocytes (*Dome-meso-EBFP2/+; srp>GFP, Rabex-5^IR^*) increased Notch expression in the MZ compared to that in controls (*Dome-meso-EBFP2/+; srp>GFP*). (C′) Expressing *Ras^V12^* in hemocytes (*Dome-meso-EBFP2/+; srp>GFP, Ras^V12^*) did not increase Notch expression in the MZ. Scale bars: 50 μm. (D) *Rabex-5* RNAi, but not *Ras^V12^*, in hemocytes (*Dome-meso-EBFP2/+; srp>GFP, Rabex-5^IR^* and *Dome-meso-EBFP2/+; srp>GFP, Ras^V12^*) decreased the percentage of lymph glands in which the MZ and CZ are discernible by Notch expression compared to that in controls (*Dome-meso-EBFP2/+; srp>GFP*). *Rabex-5* RNAi in hemocytes (*Dome-meso-EBFP2/+; srp>GFP, Rabex-5^IR^*) increased the area (E) and the Notch fluorescence intensity (F) of the primary lobes compared to those in controls (*Dome-meso-EBFP2/+; srp>GFP*). *Ras^V12^* in hemocytes (*Dome-meso-EBFP2/+; srp>GFP, Ras^V12^*) decreased the area (E) and the Notch fluorescence intensity (F) of the primary lobes compared to those in controls (*Dome-meso-EBFP2/+; srp>GFP*). Lymph glands were dissected 4 days AEL. ^*P*≤0.05, *P≤0.01.

The larval lymph gland is a site of hemocyte proliferation and differentiation with known roles for Notch signaling (Duvic et al., 2002; Lebestky et al., 2003; Krzemien et al., 2007; Small et al., 2014). The primary lymph gland lobes contain three distinct zones: the medullary zone (MZ) comprising slowly proliferating prohemocytes, the cortical zone (CZ) containing differentiating hemocytes and a small cluster of cells called the posterior signaling center (PSC), which controls the balance of prohemocytes and differentiating hemocytes. We investigated Notch dysregulation upon *Rabex-5* knockdown within the primary lobes by using an antibody that recognizes the intracellular domain of Notch (C17.9C6, DSHB). The MZ of the primary lymph gland lobes was marked using domeless-meso-EBFP2 (Fig. 5A-C,A″-C″, Table S1). In control larvae, Notch antibody staining within the MZ was moderate and uniform. This was easily discernable from intense and heterogeneous Notch antibody staining within the CZ. Thus, in 80% of control larvae, Notch expression also delineated the boundary between the MZ and CZ (Fig. 5A′,D). Reducing *Rabex-5* levels across the entire primary lobe by using *srp-gal4* (*srp>Rabex-5^IR^*) dramatically increased Notch antibody staining in the MZ (Fig. 5B′), making the MZ–CZ boundary no longer discernable by Notch expression patterns. Consequently, *Rabex-5* reduction decreased the percentage of lymph glands that display differential Notch staining between the MZ and CZ from 80% to 25% (Fig. 5D). The area (Fig. 5E), and Notch fluorescence intensity (Fig. 5F) of the entire primary lobe also increased upon *Rabex-5* reduction.

Notch and Ras demonstrate context-dependent interactions (Sundaram, 2005). To rule out a role for increased Ras activity in Notch dysregulation in the larval lymph gland, we expressed constitutively active *Ras* across the entire primary lobe (*srp>Ras^V12^*). *Ras^V12^* did not significantly alter the percentage of lymph glands displaying differential Notch staining (Fig. 5C′,D) but significantly decreased the lymph gland area (Fig. 5E). Surprisingly, *Ras^V12^* expression decreased Notch fluorescence intensity (Fig. 5F) of the primary lobe. This suggests that increased Ras activity is not sufficient to promote Notch signaling in the lymph gland.

### *Rabex-5* is required in embryonic and medullary zone hemocytes

**Fig. 6.**
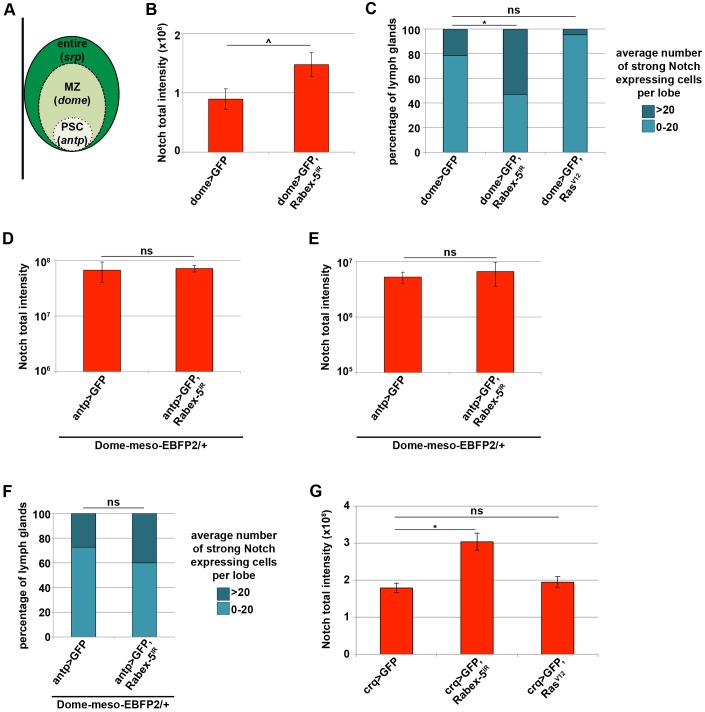
***Rabex-5* is required in the medullary zone of the larval lymph gland to restrict Notch accumulation.** (A) Schematic depicting primary lobe of the larval lymph gland. *Srp-gal4* is expressed across the entire lobe. *Dome-gal4* is expressed exclusively in the MZ. *Antp-gal4* is expressed exclusively in the PSC. (B) *Rabex-5* RNAi in the MZ (*dome>GFP, Rabex-5^IR^*) increased Notch fluorescence intensity over the entire lobe compared to that in controls (*dome>GFP*) 4 days AEL. (C) At 18°C, *Rabex-5* RNAi in the MZ (*dome>GFP, Rabex-5^IR^*) increased the average number of cells per lobe that strongly express Notch compared to those in controls (*dome>GFP*). *Ras^V12^* expression in the MZ (*dome>GFP, Ras^V12^*) did not alter the average number of cells per lobe that strongly express Notch. *Rabex-5* RNAi in the PSC (*antp>GFP, Rabex-5^IR^*) did not change Notch fluorescence intensity over the entire primary lobe (D) or in the PSC (E) compared to those in controls (*antp>GFP*). (F) *Rabex-5* RNAi in the PSC (*antp>GFP, Rabex-5^IR^*) had no effect on the average number of cells per lobe that strongly express Notch compared to those in controls (*antp>GFP*). (G) *Rabex-5* RNAi, but not *Ras^V12^*, in embryonic hemocytes (*crq>GFP, Rabex-5^IR^* and *crq>GFP, Ras^V12^*) increased Notch fluorescence intensity of the primary lobes compared to that of controls (*crq>GFP*) 5 days AEL. ^*P*≤0.05, *P≤0.01.

To determine which zone of the primary lobe required *Rabex-5* to regulate Notch, we knocked down *Rabex-5* exclusively in the MZ using *dome-gal4* or exclusively in the PSC using *antennapedia-gal4* (*antp-gal4*) (Fig. 6A, Table S1). A significant increase in Notch intensity across the entire lymph gland was seen upon RNAi of *Rabex-5* in the MZ (*dome>Rabex-5^IR^*, Fig. 6B). Per lobe, the average number of cells with strong Notch expression increased, even when larvae were raised at 18°C to minimize the effect of RNAi (Fig. 6C). Similar to *Rabex-5* reduction in the entire primary lobe, *Rabex-5* reduction exclusively in the MZ promoted Notch accumulation, supporting a model that *Rabex-5* is required to restrict Notch accumulation in the prohemocytes of the MZ. Constitutive Ras activation in the MZ had no effect on Notch expression; at 18°C, *Ras^V12^* expression by using *dome-gal4* did not significantly alter the average number of cells per lobe that strongly express Notch (*dome>Ras^V12^*, Fig. 6C).

RNAi depletion of *Rabex-5* specifically in the PSC (*antp>Rabex-5^IR^*) did not change Notch fluorescence intensity over the entire primary lobe (Fig. 6D) or in the PSC itself (Fig. 6E). RNAi of *Rabex-5* in the PSC did not increase the average number cells per lobe that strongly express Notch (Fig. 6F). These results indicate that *Rabex-5* functions in the prohemocytes of the MZ, but not in the PSC, to prevent Notch accumulation.

Given that *Rabex-5* knockdown in embryonic hemocytes did not affect crystal cell numbers (Fig. 3H), we assessed the requirement for *Rabex-5* to regulate Notch specifically in hemocytes of embryonic origin. *Rabex-5* knockdown in embryonic hemocytes (*crq>Rabex-5^IR^*) was sufficient to increase Notch fluorescence intensity across the primary lymph gland lobes. Consistent with our previous results, *Ras^V12^* expression (*crq>Ras^V12^*) did not promote Notch accumulation in the lymph gland (Fig. 6G). These data demonstrate that *Rabex-5* functions both in embryonic hemocytes and in the larval lymph gland to restrict Notch accumulation.

### Notch accumulation upon *Rabex-5* loss leads to increased transcriptional outputs

**Fig. 7.**
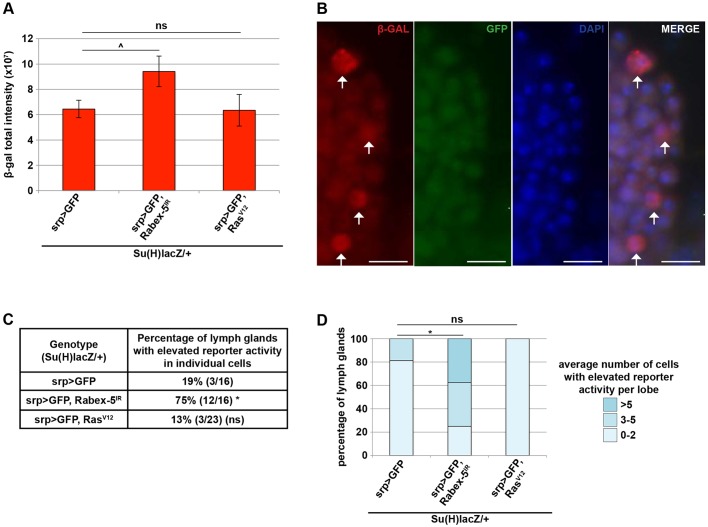
**Notch accumulation upon *Rabex-5* loss leads to increased transcriptional outputs.** (A) *Rabex-5* RNAi, but not *Ras^V12^*, in hemocytes (*srp>GFP, Rabex-5^IR^* and *srp>GFP, Ras^V12^*) increased β-gal fluorescence intensity across the primary lymph gland lobes compared to that in controls (*srp>GFP*). (B) Representative image showing individual cells with elevated reporter activity (arrows) in lymph glands expressing GFP in hemocytes. DAPI staining is shown in blue. Scale bars: 10 μm. (C) *Rabex-5* RNAi, but not *Ras^V12^*, in hemocytes (*srp>GFP, Rabex-5^IR^* and *srp>GFP, Ras^V12^*) increased the percentage of lymph glands with elevated reporter activity in individual cells compared to that of controls (*srp>GFP*). (D) *Rabex-5* RNAi, but not *Ras^V12^*, in hemocytes (*srp>GFP, Rabex-5^IR^* and *srp>GFP, Ras^V12^*) increased the average number of cells per lobe with elevated reporter activity compared to those in controls (*srp>GFP*). ^*P*≤0.05, *P≤0.01.

To establish whether Notch protein accumulation results in increased Notch transcriptional activity, we examined the effect of *Rabex-5* knockdown on a Notch transcriptional reporter, 12xSu(H)bs-lacZ (Go et al., 1998). Reducing *Rabex-5* across the primary lymph gland lobes (*srp>Rabex-5^IR^*) increased β-galactosidase (β-gal) fluorescence intensity compared to that in controls (Fig. 7A). In 81% of control lymph glands, β-gal staining was uniform and low. The remaining 19% of lymph glands showed individual cells with elevated reporter activity (arrows in Fig. 7B, quantification in 7C). *Rabex-5* reduction increased the percent of lymph glands showing individual cells with elevated reporter activity from 19% to 75%. Compared to controls, *Rabex-5* reduction also increased the average number of individual cells with elevated activity per primary lobe (Fig. 7D). These findings indicate that Notch protein accumulation upon *Rabex-5* knockdown in the lymph gland leads to functionally increased Notch transcriptional activity.

*Ras^V12^* expression (*srp>Ras^V12^*) had no effect on β-gal fluorescence intensity (Fig. 7A), did not significantly alter the percentage of lymph glands showing individual cells with elevated reporter activity (Fig. 7C), and did not significantly alter the average number of cells displaying elevated reporter activity per lobe (Fig. 7D). These results indicate that increased Ras activity is not sufficient to increase Notch transcriptional activity in the lymph gland.

### Increased Notch activity mechanistically underlies specific *Rabex-5* hematopoietic phenotypes

**Fig. 8.**
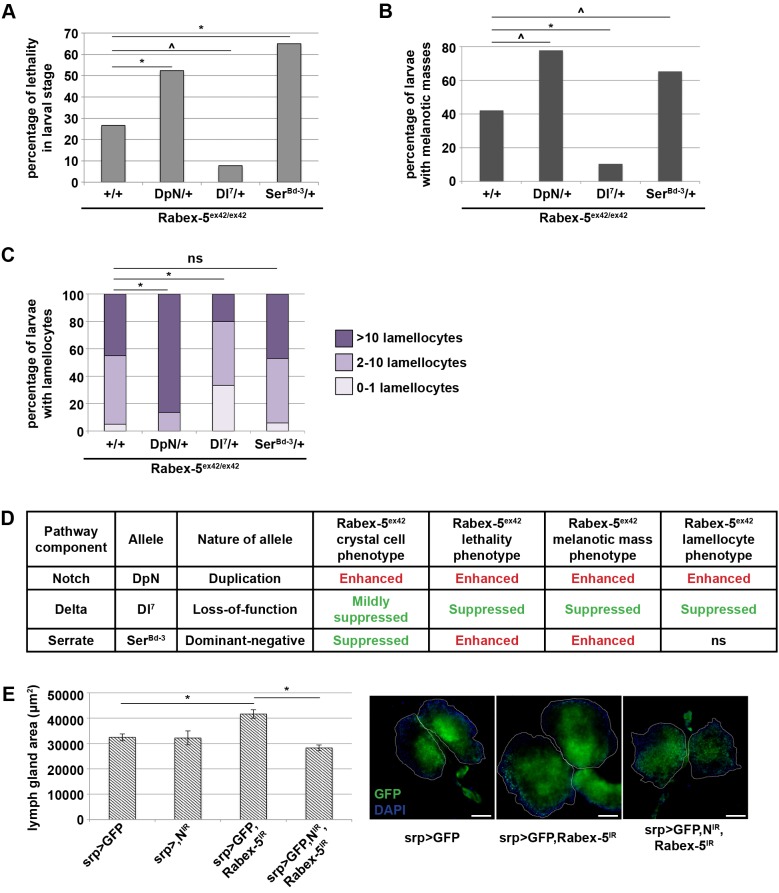
***Rabex-5* negative regulation of Notch is required for proper regulation of hematopoiesis during development.** (A) In a *Rabex-5^ex42/ex42^* background, *DpN* and *Ser^Bd-3^* (*Rabex-5^ex42/ex42^; DpN/+* and *Rabex-5^ex42/ex42^; Ser^Bd-3^/+*) increased larval lethality compared to that in controls (*Rabex-5^ex42/ex42^*). *Dl^7^* (*Rabex-5^ex42/ex42^; Dl^7^/+*) suppressed larval lethality. (B) In a *Rabex-5^ex42/ex42^* background 14 days AEL, *DpN* and *Ser^Bd-3^* (*Rabex-5^ex42/ex42^; DpN/+* and *Rabex-5^ex42/ex42^; Ser^Bd-3^/+*) increased the incidence of melanotic masses and *Dl^7^* (*Rabex-5^ex42/ex42^; Dl^7^/+*) decreased the incidence of melanotic masses compared to those in controls (*Rabex-5^ex42/ex42^*). (C) In a *Rabex-5^ex42/ex42^* background 6 days AEL, *DpN* (*Rabex-5^ex42/ex42^; DpN/+*) increased the percentage of larvae with lamellocytes compared to that in controls (*Rabex-5^ex42/ex42^*). *Dl^7^* (*Rabex-5^ex42/ex42^; Dl^7^/+*) decreased the percentage of larvae with lamellocytes. *Ser^Bd-3^* (*Rabex-5^ex42/ex42^; Ser^Bd-3^/+*) did not alter the percentage of larvae with lamellocytes compared to that in controls (*Rabex-5^ex42/ex42^*). (D) Summary of *Rabex-5^ex42/ex42^* genetic interactions with *Notch*, *Delta* and *Serrate*. (E) *Notch* RNAi in hemocytes (*srp>GFP, N^IR^*) did not affect the area of the primary lymph gland lobes at 21°C but reduced the enlarged lymph glands (*srp>GFP, N^IR^, Rabex-5^IR^*) resulting from *Rabex-5* RNAi (*srp>GFP, Rabex-5^IR^*) to control area (*srp>GFP*) 7 days AEL. DAPI staining is shown in blue. Scale bars: 50 μm; ^*P*≤0.05, *P≤0.01.

To establish whether the increased Notch activity is functionally relevant to *Rabex-5* hematopoietic phenotypes, we performed genetic interactions by using the Notch pathway components *Notch*, *Delta* and *Serrate* (Fig. 8A-C, summarized in 8D), or performing RNAi of *Notch* (Fig. 8E). Reducing *Delta* gene dosage using the *Dl^7^* allele suppressed larval lethality in a *Rabex-5*-null background. *Notch* duplication (*DpN*) enhanced larval lethality. Surprisingly, larval lethality was also enhanced by the *Ser^Bd-3^* allele (Fig. 8A,D), which produces a protein that lacks the intracellular and transmembrane domains but retains the Notch binding domain (Hukriede and Fleming, 1997). If this truncated Serrate is able to activate Notch in some contexts, it might modify *Rabex-5*-null phenotypes similar to *Notch* duplication. Similarly, *Dl^7^* suppressed the *Rabex-5*-null melanotic mass phenotype, whereas *DpN* and *Ser^Bd-3^* enhanced the phenotype (Fig. 8B,D). *Dl^7^* suppressed lamellocyte differentiation in *Rabex-5*-null larvae, *DpN* dramatically enhanced lamellocyte differentiation, and *Ser^Bd-3^* had no effect (Fig. 8C,D). RNAi of *Notch*, by using the inducible inverted-repeat allele *N^IR^* in hemocytes (*srp>N^IR^*), did not affect the size of the lymph gland at 21°C but suppressed the increased lymph gland area resulting from *Rabex-5* knockdown (*srp>GFP, Rabex-5^IR^* and *srp>GFP, N^IR^, Rabex-5^IR^*, Fig. 8E). These results indicate that increased Notch activity contributes to larval lethality and is functionally relevant to the melanotic mass, lamellocyte differentiation and lymph gland size phenotypes. These data and our earlier findings are consistent with a model that *Rabex-5* regulates not only Ras activity (Yan et al., 2010) but also Notch activity in a Ras-independent manner during hematopoiesis to ensure proper restriction of hemocyte proliferation, to direct or prevent differentiation into specific lineages, and to maintain hematopoietic homeostasis.

## DISCUSSION

We report a requirement for *Rabex-5* to ensure proper hematopoiesis in *Drosophila*. *Rabex-5*-null mutants exhibited a range of hematopoietic abnormalities, including hemocyte overproliferation, increased lymph gland size, increased crystal cell populations, lamellocyte differentiation and melanotic mass formation. *Rabex-5* is a known *Drosophila* neoplastic tumor suppressor (Yan et al., 2010; Thomas and Strutt, 2014); inactivating mutations in *Rabex-5* cause tissue overgrowth and extend larval development. Immune responses to overgrown tissue have been demonstrated in both *Drosophila* (Pastor-Pareja et al., 2008; Hauling et al., 2014) and mammalian systems (Hanahan and Weinberg, 2011). We cannot exclude that an immune response to overgrowing tissue or a prolonged larval period partly contributes to *Rabex-5*-null phenotypes. However, restoring wild-type *Rabex-5* activity in hemocytes suppressed melanotic mass formation, and reducing *Rabex-5* in the hematopoietic system – which does not delay development – was sufficient to reproduce almost all *Rabex-5*-null phenotypes. This indicates a role for *Rabex-5* specifically in the hematopoietic system. Interestingly, a *Rabex-5*-knockout mouse model shows skin inflammation, increased mast cell numbers and perinatal lethality. Bone-marrow-cultured mast cells (BMCMCs) derived from *Rabex-5*-knockout mice show enhanced and prolonged activation upon stimulation compared to that of wild-type control BMCMCs (Tam et al., 2004). These similarities suggest that the function of *Rabex-5* within the hematopoietic system is conserved in mammals.

We provide evidence that *Rabex-5* restricts both Ras and Notch signaling in order to establish proper lymph gland size and to promote organismal survival. Reducing Ras or Notch activity, as well as restricting hemocyte proliferation, suppressed the increased lymph gland size and larval lethality that results from *Rabex-5* loss. This suggests that *Rabex-5* restricts proliferation of hemocytes by downregulating Ras, consistent with reports that excess Ras signaling causes overproliferation of hemocytes (Asha et al., 2003; Zettervall et al., 2004), and also by downregulating Notch. Surprisingly, melanotic mass formation was dependent upon hemocyte proliferation and increased Notch activity but not increased Ras activity. In this context, *Rabex-5* might restrict hemocyte proliferation through a distinctively Notch-mediated mechanism. Consistent with the requirement for Notch, but not Ras, activity in crystal cell specification, the increase in crystal cells observed upon *Rabex-5* loss was dependent upon increased activity of Notch but not Ras. Additionally, increased Notch, but not increased Ras, activity was relevant to the *Rabex-5*-null lamellocyte phenotype, indicating a function for *Rabex-5* to regulate Notch during hemocyte specification. All together, these results are consistent with a role for *Rabex-5* to restrict both Ras and Notch signaling in hemocytes.

Convergence of the Ras and Notch pathways is required for specification of blood progenitors in the *Drosophila* embryo (Grigorian et al., 2011b). Importantly, we provide evidence that *Rabex-5* does not regulate Notch through its regulation of Ras in the hematopoietic system. Constitutively active Ras expression did not phenocopy the effect of *Rabex-5* loss in the lymph gland and was insufficient to induce phenotypes associated with Notch pathway dysregulation, such as increased crystal cell populations. Few specific regulators have been identified in any system as links between these two pathways. We identify *Rabex-5* as a modulator of both Ras and Notch activity to ensure hematopoietic homeostasis; this dual role might implicate *Rabex-5* as a nexus coordinating activity of these pathways, and raises interesting questions regarding the spatiotemporal regulation of Ras and Notch by *Rabex-5* specifically in the hematopoietic system and, more generally, in developmental contexts that require Ras and Notch interplay.

To this point, we reveal a spatiotemporal requirement for *Rabex-5* during the two waves of *Drosophila* hematopoiesis. Reducing *Rabex-5* in hemocytes specifically of embryonic or of larval origin was sufficient to increase lymph gland size and Notch accumulation. *Rabex-5* reduction in larval, but not embryonic, hemocytes increased crystal cell numbers. *Rabex-5* reduction in both embryonic and larval hemocytes, but not in embryonic hemocytes alone, was sufficient to induce melanotic masses. These findings demonstrate an intrinsic requirement for *Rabex-5* in the hematopoietic system with overlapping and distinct roles during embryonic and larval hematopoiesis.

Excitingly, our data might implicate *Delta* in *Drosophila* hematopoiesis. Except in the control of blood progenitor specification and proliferation in the embryo (Mandal et al., 2004; Grigorian et al., 2011b), *Delta* has not been demonstrated to function in *Drosophila* hematopoietic processes. Rather, *Serrate* is the primary ligand that activates Notch during hematopoiesis. Notch activation through *Serrate* is required for crystal cell formation (Duvic et al., 2002; Lebestky et al., 2003; Mandal et al., 2007; Krzemien et al., 2010), maintains PSC identity (Lebestky et al., 2003; Krzemien et al., 2007) and prevents lamellocyte differentiation (Small et al., 2014). We show that the reduction of *Delta* gene dosage in *Rabex-5*-null larvae suppressed lethality and melanotic masses, both of which are phenotypes that depend on hemocyte proliferation. A dominant-negative *Serrate* allele suppressed the increase in crystal cell numbers in a *Rabex-5*-null background, whereas any suppression mediated by the reduction of *Delta* was subtle. One interpretation of these findings is that Notch activation via *Delta* affects hemocyte proliferation, whereas Notch activation through *Serrate* affects hemocyte differentiation.

Our findings have implications for human disease. In mammals, Notch controls decisions of multipotent hematopoietic cells to self-renew, proliferate, commit and differentiate to specific lineages. The importance of Notch in mammalian hematopoiesis is emphasized by the frequency of Notch alterations in human hematological malignancies, including leukemia. Excitingly, we identify *Rabex-5* as an important regulator of Notch in the prohemocytes of the larval lymph gland. Prohemocytes most closely resemble the mammalian common myeloid progenitor, and evidence for Notch involvement in myeloid leukemias is emerging. Sequencing of acute myeloid leukemias (AMLs) revealed that two-thirds of AML cases in which *Rabex-5* mRNA is downregulated show upregulation of *Notch*, *Delta* or *Jagged2* (a mammalian *Serrate* ortholog) mRNA (Cerami et al., 2012; The Cancer Genome Atlas Research Network, 2013; Gao et al., 2013). However, there are conflicting reports on the role of Notch signaling in AMLs, which might reflect unresolved heterogeneity within this cancer type (Tohda and Nara, 2001; Asha et al., 2003; Tohda et al., 2005; Kannan et al., 2013; Zhang et al., 2013). The status of *Rabex-5* might help to further define subsets of AML, and might provide tremendous opportunities to elucidate the etiology – and inform on the treatment – of human leukemia.

## MATERIALS AND METHODS

### Drosophila

Flies were raised at 25°C on standard medium unless otherwise stated. Fly genotypes are listed in Table S2.

### Larval staging and quantification

For lethality, melanotic mass and lamellocyte quantification, flies were permitted to lay eggs for 1 day. Control larvae were evaluated 6 days after egg laying (AEL), and experimental larvae were evaluated 6, 9 or 14 days AEL. For circulating hemocyte quantification, flies were permitted to lay eggs for 2 h. Control larvae were evaluated 120 h AEL, and experimental larvae were evaluated 120 h or 9 days AEL. Hemolymph from individual larva was collected by tearing open and inverting the cuticle in 5 μl drops of phosphate-buffered saline (PBS) on coverslips pre-treated with poly-D-lysine. Hemolymph was fixed with 5 μl of 7.5% paraformaldehyde in PBS for 15 min at room temperature (RT) and washed three times with PBS. Cells were permeabilized with 0.1% Tween-20 in PBS with 5% either natural goat serum, natural donkey serum or bovine serum albumin (BSA) for 20 min at RT. Hemocytes were counted from at least five random frames per individual larva. The percentage of hemocytes, plasmatocytes and crystal cells was calculated by dividing the number of GFP-positive, P1a/P1b-positive, lozenge-positive or melanized cells by the number of DAPI-positive cells.

### Circulating hemocyte concentrations

Flies were permitted to lay eggs for 2 h. Control larvae were evaluated 120 h AEL. Experimental larvae were evaluated 120 h, 9 days and 14 days AEL. Hemolymph from each individual larva was collected in 20 μl of PBS and kept on ice. Hemocyte concentration was measured in cells/ml using a Countess Automated Cell Counter from Invitrogen and plotted as relative concentration. Minimum and maximum cell sizes were set to 2 μm and 22 μm, respectively, and circularity was restricted to 75-80% roundness.

### Crystal cell melanization

Flies were permitted to lay eggs for 1 day. Third instar larvae were collected and washed in PBS, dried and individually placed in PCR tubes. Larvae were heated at 60°C for 10 min in an Eppendorf Mastercycler EP Gradient S thermal cycler to induce melanization of crystal cells. Two lab members blindly scored larvae.

### Lymph gland preparations

Flies were permitted to lay eggs for 6 h. Lymph glands were dissected as described in standard protocols (Evans et al., 2014) 4 or 5 days AEL, fixed with 3.7% paraformaldehyde in PBS for 30 min on ice, washed three times with PBS, and permeabilized in PBS with 0.1% Tween-20 and 5% BSA for 20 min at RT. Lymph glands were incubated in antibodies (see below) and mounted according to standard protocols (Evans et al., 2014).

### Immunohistochemistry

Antibodies were diluted in 0.1% Tween-20 in PBS with 5% natural goat serum, natural donkey serum or BSA. P1a/1b and L1a/L1b/L1c antibody mixtures were diluted 1:750 (István Andó, Hungarian Academy of Sciences, Szeged, Hungary). Antibodies used were: Lozenge antibody at 1:20 (anti-lozenge, DSHB), Notch intracellular domain antibody at 1:500 (C17.9C6, DSHB), β-galactosidase antibody at 1:1000 (G4644, Sigma), Alexa-Fluor-555 goat anti-mouse HCA secondary antibody at 1:2000 (Invitrogen). Incubations were overnight at 4°C for primary antibodies and at least 2 h at RT for secondary antibodies.

### Microscopy

Larvae with melanotic masses (Fig. 1A) and melanized crystal cells (Figs 2G, 3H,J and 4H, Fig. S2F) were imaged with a Nikon SMZ1000 stereomicroscope. Still frames from movies of live larvae with GFP-labeled hemocytes (Fig. 2A) were taken with a Zeiss Axio Observer.Z1. Fixed hemocytes (Fig. 2E) were imaged with a Zeiss Axio Imager.Z1. Z-stacks of lymph glands were taken with a Zeiss Axio Imager.Z1 and analyzed using Zen software. Regions of interest (ROIs) surrounding the primary lymph gland lobes and 21 Z-positions (9.8 μm) surrounding the center Z-position were selected. With the exception of lymph glands marked with Trol (Fig. 2B, Fig. S3), constrained iterative deconvolution was applied to all lymph gland images prior to analysis of primary lobes (Figs 3B, 5A-F, 6B-G, 7A-D and 8E, Fig. S2A). All lymph gland images are presented as a single maximum-intensity projection.

### Statistical analysis

Student’s unpaired *t*-tests compared lymph gland areas, hemocyte concentrations and fluorescence intensities. Error bars represent ±s.e.m. Chi-square tests compared percentages of larvae with melanotic masses, larval lethality, hemocyte percentages, lamellocyte percentages and lymph gland percentages. Statistically significant *P* values are indicated in the figure panels; ^*P*≤0.05 and *P≤0.01. For lamellocyte experiments, *n*≥6 larvae of each genotype were scored. For quantification of hemocyte numbers, *n*≥6 larvae of each genotype were scored. For lymph gland area measurements, *n*≥6 larvae of each genotype were scored. For fluorescence intensity measurements, *n*≥8 larvae of each genotype were scored. For heat-induced melanization experiments, *n*≥11 larvae of each genotype were scored. For larval lethality experiments, *n*≥20 larvae of each genotype were scored. For melanotic mass experiments: *n*≥20 with the exception of flies carrying *DpN*, where *n*=9. Data shown are representative results from reproducible experiments.
